# Structures of the Inducer-Binding Domain of Pentachlorophenol-Degrading Gene Regulator PcpR from *Sphingobium chlorophenolicum*

**DOI:** 10.3390/ijms151120736

**Published:** 2014-11-12

**Authors:** Robert P. Hayes, Timothy W. Moural, Kevin M. Lewis, David Onofrei, Luying Xun, ChulHee Kang

**Affiliations:** 1 Department of Chemistry, Washington State University, Pullman, WA 99164-4630, USA; E-Mails: robert_hayes@wsu.edu (R.P.H.); timothy.moural@email.wsu.edu (T.W.M.); kevin.lewis@email.wsu.edu (K.M.L.); 2 School of Molecular Biosciences, Washington State University, Pullman, WA 99164-4660, USA; E-Mail: david.onofrei@email.wsu.edu

**Keywords:** LysR-family, transcription factor, bioremediation, pentachlorophenol, 2,4,6-trichlorophenol

## Abstract

PcpR is a LysR-type transcription factor from *Sphingobium chlorophenolicum* L-1 that is responsible for the activation of several genes involved in polychlorophenol degradation. PcpR responds to several polychlorophenols *in vivo*. Here, we report the crystal structures of the inducer-binding domain of PcpR in the apo-form and binary complexes with pentachlorophenol (PCP) and 2,4,6-trichlorophenol (2,4,6-TCP). Both X-ray crystal structures and isothermal titration calorimetry data indicated the association of two PCP molecules per PcpR, but only one 2,4,6-TCP molecule. The hydrophobic nature and hydrogen bonds of one binding cavity allowed the tight association of both PCP (*Kd* = 110 nM) and 2,4,6-TCP (*Kd* = 22.8 nM). However, the other cavity was unique to PCP with much weaker affinity (*Kd* = 70 μM) and thus its significance was not clear. Neither phenol nor benzoic acid displayed any significant affinity to PcpR, indicating a role of chlorine substitution in ligand specificity. When PcpR is compared with TcpR, a LysR-type regulator controlling the expression of 2,4,6-trichlorophenol degradation in *Cupriavidus necator* JMP134, most of the residues constituting the two inducer-binding cavities of PcpR are different, except for their general hydrophobic nature. The finding concurs that PcpR uses various polychlorophenols as long as it includes 2,4,6-trichlorophenol, as inducers; whereas TcpR is only responsive to 2,4,6-trichlorophenol.

## 1. Introduction

**Figure 1:**
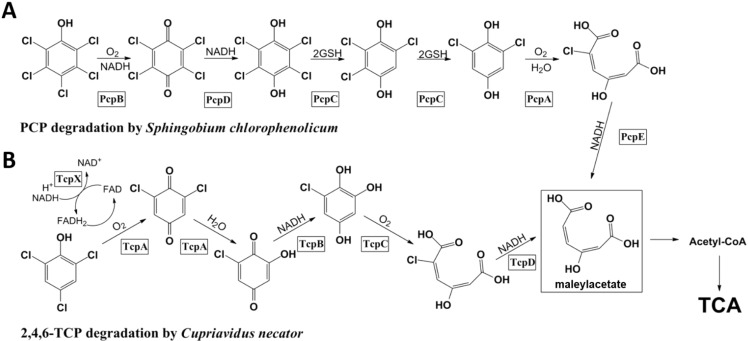
Degradation pathway of pentachlorophenol (PCP) in *Sphingobium chlorophenolicum* and 2,4,6-TCP in *Cupriavidus necator*. (**A**) PCP is converted to maleylacetate through a series of enzymatic transformations. PcpR is responsible for the transcriptional control of PcpA, PcpB and PcpE; and (**B**) 2,4,6-TCP is converted to maleylacetate through a series of enzymatic transformations. TcpR is responsible for the transcriptional control of TcpX, TcpA, TcpB, TcpC and TcpD.

The mechanism of microbial degradation of polychlorophenols has been extensively studied over the past several decades with the goal of developing effective bioremediation strategies. Accordingly, genetic organization, biochemical characterization and structural analysis have been conducted for the enzymes involved in the degradation of pentachlorophenol (PCP) in *Sphingobium chlorophenolicum* L-1 [1,2,3,4,5,6] and 2,4,6-trichlorophenol (2,4,6-TCP) in *Cupriavidus necator* JMP134 [7,8,9]. In *S. chlorophenolicum*, PCP is converted to maleylacetate by the concerted actions of a monooxygenase (PcpB) [3], a quinone reductase (PcpD) [3,5], a reductive dechlorinase (PcpC) [10], a ring cleavage 1,2-dioxygenase (PcpA) [1,2,6], and a chloromaleylacetate reductase (PcpE) [4]. Maleylacetate is further channeled to the tricarboxylic acid (TCA) cycle for complete mineralization (Figure 1A). PcpR is responsible for the transcriptional control of PcpA, PcpB, and PcpE, however, PcpC is constitutively produced [4]. The degradation pathway of 2,4,6-TCP to chloromaleylacetate in *C. necator* involves a two-component flavin-diffusible monoxygenase system (TcpX and TcpA), a quinone reductase (TcpB), a ring-cleaving dioxygenase (TcpC), and a chloromaleylacetate reductase (TcpD) [7]. The produced maleylacetate is also channeled to the TCA cycle for complete mineralization. TcpR is responsible for the transcriptional control of all these proteins (Figure 1B) [8].

Understanding transcriptional control of these genes by PcpR and TcpR is critical for the development of bioremediation strategies. PcpR and TcpR belong to the LysR transcriptional regulator (LTTR) and are responsible for controlling the degradation of PCP and 2,4,6-TCP, respectively, although their precise mechanism of transcriptional control is not well defined. The LysR-type regulators have also been shown to control gene expression for the degradation of other aromatic compounds, including benzoate and chlorinated aromatic compounds [11]. In general, the LysR family transcription factors consist of an *N*-terminal DNA-binding domain (DBD) and a *C*-terminal inducer-binding domain (IBD) [12,13,14,15].

*S. chlorophenolicum* uses the PCP pathway (Figure 1A) to degrade a variety of polychlorophenols as long as both the 2- and 6-positions of the phenol rings are substituted with chlorinesand [3] (Xun and Orser 1991). Conversely, *C. necator* JMP134 can only degrade 2,4,6-TCP [8] (Sánchez and González, 2007). The difference is also reflected in the specificities of PcpR and TcpR. PcpR is activated by several polychlorophenols including PCP, 2,3,5,6-tetrachlorophenol (TeCP), 2,4,6,-trichlorophenol (TCP) and 2,6-dichlorophenol (2,6-DiCP) [3,4] (Xun and Orser 1991). In contrast, TcpR is activated by only 2,4,6-TCP [8]. The noticeable overlap and difference of substrate specificity are likely due to specific amino acid substitutions in the inducer-binding pocket. 

Proper transcriptional control of the participating genes is crucial for *in situ* bioremediation. Thus, in order to maximize the efficiency of biodegradation, in-depth knowledge of the regulation mechanisms is required. In this report, we present crystal structures of the PcpR inducer-binding domain in apo-form and in complex with the native inducers, PCP and 2,4,6-TCP. Structural information was coupled with the thermodynamic analysis of inducer binding by isothermal titration calorimetry (ITC), provides a clear understanding for specificity differences and which helps to shed light on the overall transcriptional regulation mechanism by PcpR and TcpR and [12,13,14,16,17,18].

## 2. Results and Discussion

### 2.1. Global and Oligomeric Structure of PcpR

**Figure 2:**
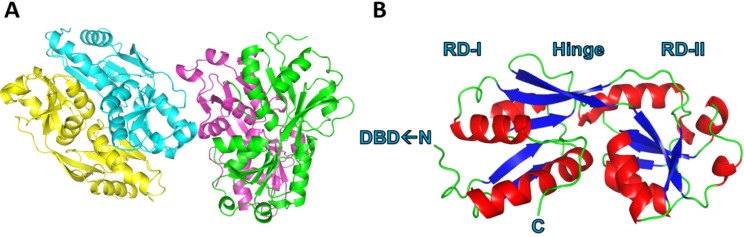
Global structure of PcpR. (**A**) Ribbon diagram representing two PcpR dimers in the asymmetric unit; and (**B**) Overall global structure of PcpR monomer illustrating the two Rossmann-like domains RDI and RDII, the hinge region, and the *C*-terminal and *N*-terminal of this construct (labelled DBD←N and C respectively). This figure was generated using the PyMOL Molecular Graphics System, Version 1.3, Schrödinger, LLC (Cambridge, MA, USA).

**Figure 3:**
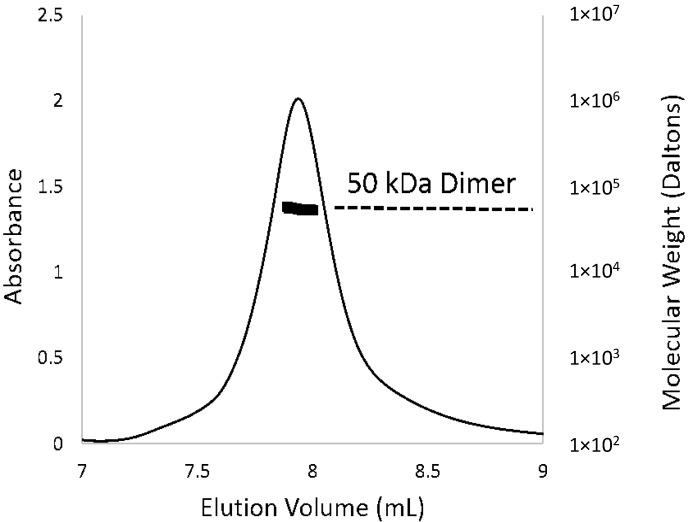
Oligomeric state of PcpR in solution. The elution profile for PcpR was monitored with multi-angle laser light scattering and was shown as absorbance (left-*Y* axis) and molecular weight (right-*Y* axis) *versus* elution volume (mL). The solid line represents changes in absorption at 280 nm. The thick black cluster in the middle of the peak indicated the calculated molecular mass (~50 kD) from the light scattering illustrating the dimeric nature of PcpR.

Purified recombinant PcpR protein, which covered the substrate-binding domain (residues from 85 to the *C*-terminus of PcpR), was crystallized in the R3 space group with 4 molecules per asymmetric unit (Figure 2A) and solved at 2.7, 2.2 and 1.9 Å resolution for the apo-form, PCP and 2,4,6-TCP binary complexes, respectively (Table 1). The monomeric structure of the PcpR inducer-binding domain contains two nucleotide-binding (or Rossmann fold) motifs connected by a hinge region (Figure 2B). Although 4 molecules were seen in the asymmetric unit, PcpR appears to exist as dimers in solution as illustrated by multi-angle light scattering (MALS) (Figure 3) consistent with weak interaction between the two dimers in the crystal lattice. The *N*-, *C*-terminus and two loop regions (173–177 and 183–186) of the four molecules in the asymmetric unit and among three structures showed somewhat different conformations indicating their flexible nature.

**Table 1:** Data collection and refinement statistics.

	PcpR-Apo	PcpR-PCP	PcpR-TCP
**Data Collection**			
Space group	R3	R3	R3
Cell dimensions			
*a*, *b*, *c* (Å)	169.45, 169.45, 109.84	169.05, 169.05, 110.26	169.75, 169.75, 110.15
α, β, γ (°)	90.00, 90.00, 120.00	90.00, 90.00, 120.00	90.00, 90.00, 120.00
Resolution (Å)	50.00–2.70 (2.80–2.70) *	50.00–2.27 (2.31–2.27) *	50.00–1.94 (1.98–1.94) *
*R*_sym_	0.139 (0.448) *	0.082 (0.346) *	0.080 (0.810) *
*I*/σ*I*	13.99 (0.77) *	18.50 (2.34) *	14.64 (2.18) *
Completeness (%)	98.6 (85.9) *	99.1 (91.9) *	100.0 (100.0) *
Redundancy	5.4 (3.5) *	5.5 (3.4) *	5.7 (5.6) *
**Refinement**			
Resolution (Å)	42.36–2.70	49.46–2.27	50.00–1.95
No. reflections	57389	53656	86296
*R*_work_/*R*_free_	0.1605/0.2159	0.1767/0.2151	0.1775/0.2093
No. atoms			
Protein	6901	6995	7006
Ligand/ion	0	96	56
Water	112	343	565
*B*-factors			
Protein	45.70	38.40	35.80
Ligand/ion	N/A	35.70	35.90
Water	43.00	42.10	42.80
**Root mean square deviations**			
Bond lengths (Å)	0.010	0.009	0.009
Bond angles (°)	1.09	1.02	1.14
Protein Data Bank ID	4RNS	4RPN	4RPO

* Values in parentheses are for highest-resolution shell.

The PcpR dimer was established by interactions between β-strands and α-helices from each monomer. Two pairs of α-helices, related by a two-fold axis, formed a hydrophobic dimer interface. Residues participating in the hydrophobic interactions at this α-helix interface include F106, A109, L112, P113, V233, V236 and I237. The β-strand of residues 127–129 formed three inter-dimer hydrogen bonds to the backbone residues 225–227 of the adjacent monomer, establishing two continuous β-sheets spanning through the two monomers in a two-fold manner. Salt bridges were also observed between residues enforcing a dimer interaction; such as E120 and R195 along with R225, R127 with the carbonyl of P221, and E229 with R134.

### 2.2. Inducer-Binding Cavities (IBC) in PcpR

**Figure 4:**
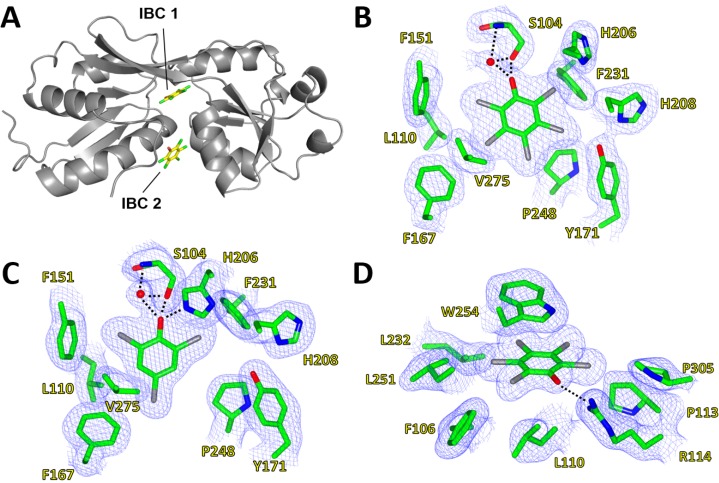
Inducer-binding cavities (IBC) of PcpR. (**A**) Relative locations of IBC1 and IBC2; (**B**) PCP binding cavity 1. 2F_o_–F_c_ map showing coverage of PCP in IBC1 under the hinge between RDI and RDII; (**C**) 2,4,6-TCP binding cavity. 2F_o_–F_c_ map showing coverage of 2,4,6-TCP in IBC1; and (**D**) PCP binding cavity 2. 2F_o_-F_c_ map showing coverage of PCP in IBC2 between α1 of RD1, α9 of RD2 and a flexible loop contributed by the adjacent monomer. These figures were generated using CCP4MG Version 2.9.0 [19].

The complex structure of PcpR with PCP displayed the clear electron density for two bound PCP molecules located in two inducer binding cavities (IBC) (Figure 4A). The first cavity (IBC1) was located underneath a hinge region between two lobes of the inducer-binding domain and the nearby second cavity (IBC2) was rather exposed to the surface. In IBC1, the PCP molecule was confined in a relatively hydrophobic pocket formed by residues S104, F106, L110, F151, F167, Y171, H206, H208, F231, P248, and V275 (Figure 4B). The crystal structure of PcpR-2,4,6-TCP complex displayed only one molecule of 2,4,6-TCP residing at IBC1, with no second molecule binding in IBC2. The 2,4,6-TCP molecule in IBC1 (Figure 4C) was bound in a similar manner to PCP, although the angular orientation of the two ligand molecules were slightly different. The root-mean square distances (rmsd) between the apo-form structure and the binary complex structures were 0.53 and 0.58 Å for PcpR-PCP complex and PcpR-2,4,6-TCP complex, respectively, indicating that there were no major backbone conformational changes upon ligand association. The relatively low *pKa* values of PCP (4.74) and 2,4,6-TCP (6.1) suggest that both ligands are likely in their phenolate form in the crystallization conditions. The oxyanion moieties of PCP and 2,4,6-TCP were 2.5 Å from the hydroxyl oxygen of S104 forming a hydrogen bond (Figure 4B,C). A water molecule was located in all apo-form and complex structures of PcpR, forming a hydrogen bond with the backbone amide nitrogen of S104 (Figure 4B,C). The oxyanion moiety of 2,4,6-TCP was hydrogen bonded with the H206-Nε atom of the imidazole ring, in addition to S104 and a water molecule. In contrast, the imidazole ring of H206 was pushed away in the PCP complex structure and not involved in a hydrogen bond network probably due to steric adjustment for the bulkier PCP.

The second binding cavity (IBC2), which was unique to the PCP complex, was located near the dimer interface between α1 of RDI and α9 of RDII. The adjacent interacting monomer also contributes its α8 helix and a flexible loop to the second PCP-binding pocket. The pocket is formed from residues F106, L110, R114, W254 and P305. PCP interacts with the indole sidechain of W254 through π-stacking, and the oxyanion of PCP interacts electrostatically with the guanidinium sidechain of R114 (Figure 4D). Additionally, two residues from the adjacent monomer at the dimer interface, V236 and E240, contributed to the second PCP-binding cavity.

### 2.3. Isothermal Titration Calorimetry (ITC) Data

**Figure 5:**
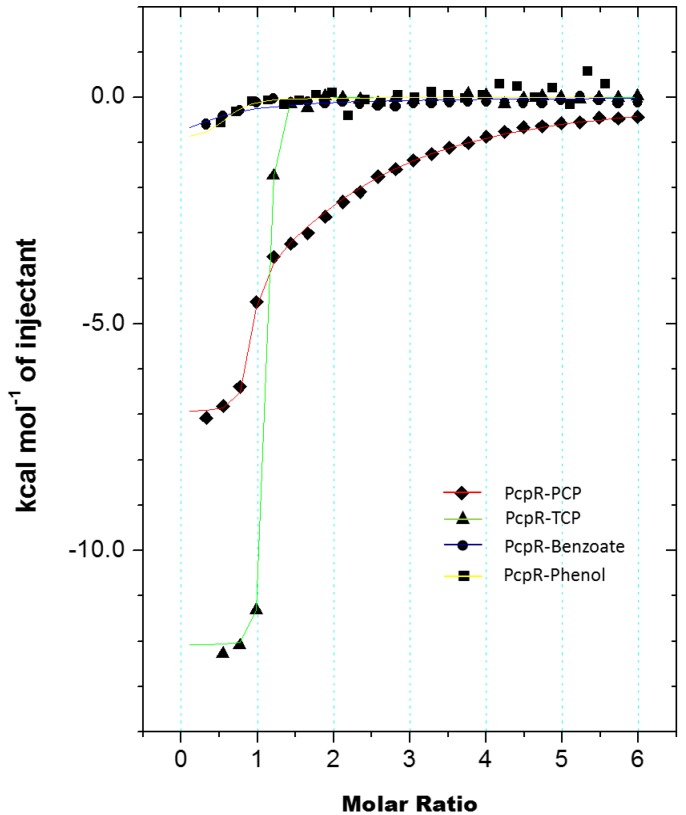
Measurement of PcpR inducer binding through isothermal titration calorimetry (ITC) experiments. The trend of heat released by serial injections of PCP, 2,4,6-TCP, sodium benzoate, and phenol. The data shows two sites-binding mode for PCP, one sites-binding mode for 2,4,6-TCP, and no significant association of either benzoate or phenol.

ITC was employed to characterize the thermodynamics of inducer-binding to PcpR. The *C*-terminal inducer-binding domain was titrated with PCP (Figure 5) and 2,4,6-TCP (Figure 5). The titration curve of PCP displayed a two-binding site mode, one tight and the other much weaker. *Kd_1_* of PCP was 110 nM and had an enthalpic contribution, ∆*H* = −6.93 kcal/mol, and a positive entropy, ∆*S* = 8.60 cal·mol^−1^·K^−1^. *Kd_2_* of PCP was substantially higher at 70 μM and had a significant enthalpic contribution, ∆*H* = −8.96 kcal/mol, and a negative entropy, ∆*S* = −11.0 cal·mol^−1^·K^−1^. Titration with 2,4,6-TCP had a single-binding mode with a *Kd* value of 22.8 nM, an enthalpic contribution of −11.9 kcal/mol, and ∆*S* = −5.04 cal·mol^−1^·K^−1^. Both phenol and benzoic acid, of which *pKa* values are 10 and 4.2 respectively, did not display any apparent affinity to PcpR (Figure 5).

### 2.4. Comparison with Other LysR-Type Transcription Factors

**Figure 6:**
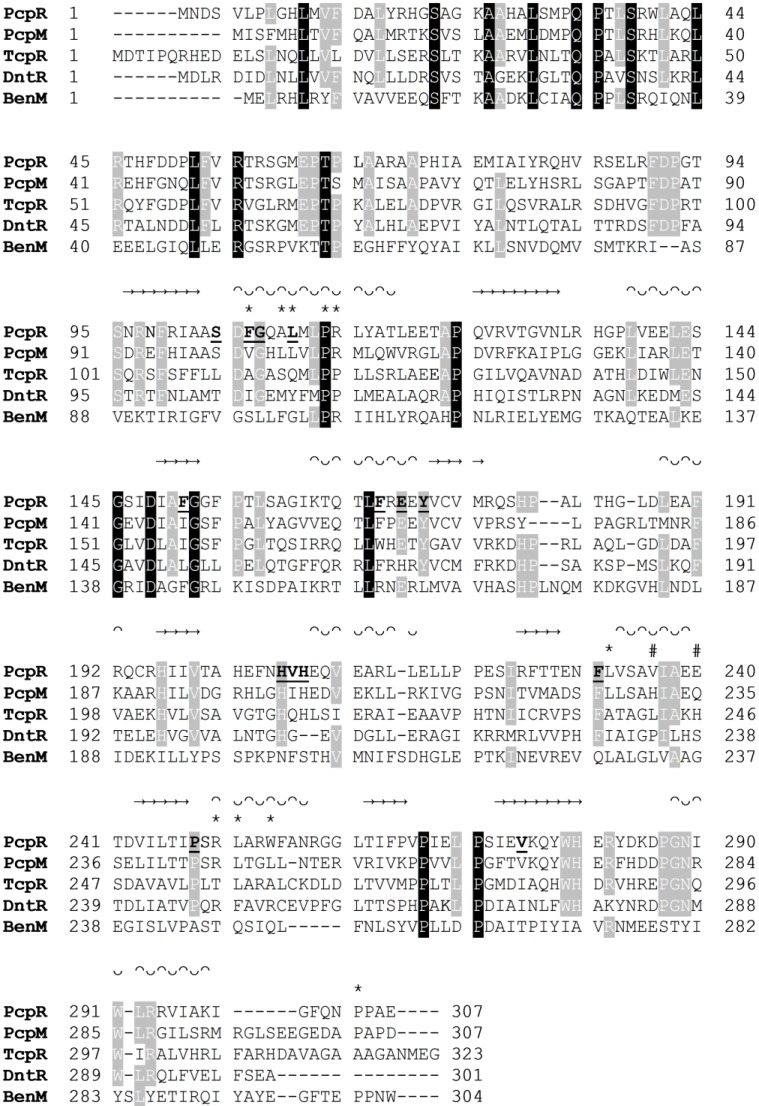
Multiple sequence alignment of PcpR, PcpM, TcpR, DntR and BenM. β-strands are signified with → and helices with ᴖᴗ. IBC1 interacting residues are marked with bolded and underlined residue symbol. IBC2 interacting residues are marked with an *. IBC2 interacting residues at the dimer interface are marked with #. Multiple sequence alignment was performed with CLUSTALW [22].

To identify PcpR homologs, a Dali search [20] was conducted with available structures in the Protein Data Bank (PDB). The most similar structure was DntR [13], a LysR-type regulator from *Burkholderia Sp* (PDB ID: 2Y7K) [13] with a high *Z* score of 25.7, followed by two putative transcriptional regulators from *Pseudomonas aeruginosa* (PDB ID: 2ESN) and *Vibrio parahaemolyticus* (PDB ID: 3OXN) with *Z* scores of 22.2 and 20.3, respectively. In addition, a BLAST [21] search of the NCBI protein data base revealed that the highest match is TcpR with 37% sequence identity followed by DntR with 34%. Sequence alignment also indicated that the residues constituting the DNA-binding and linker domain are more conserved, relative to the ligand-binding domain, among those proteins (Figure 6). This suggests that although the overall scaffold of the ligand-binding domain has been conserved, specific binding has evolved through intense mutations of binding cavities. 

The overall fold of PcpR was similar to not only DntR [13], but also another structurally established LysR-type protein, BenM, a LysR-type regulator from *Burkholderia cepacia* R34. The core rmsd between PcpR and BenM is 4.8 Å and the core rmsd between PcpR and DntR is ~1.5 Å. Further comparison with the inducer-bound complex structures of BenM-benzoate (PDB ID: 2F78) and DntR-salicylate (PDB ID: 2Y7K) illustrated that all three proteins share approximately the same location for IBC1. Benzoate and salicylate were 3.6 and 4.0 Å, respectively, separated from the spatial center of the PCP molecule in IBC1 of PcpR. However, the location of IBC2 in these proteins is drastically different [13].

Although the polar residues are not conserved, the hydrophobic residues constituting IBC1 in PcpR are somewhat conserved among TcpR, DntR, and BenM (Figure 6). When specifically comparing IBC1 of PcpR with that of TcpR, the PcpR residues S104, L110, F151, F167, V207 and V275 are replaced by L110, Q116, I157, W173, Q213 and I281. In particular, the sidechain of S104 established a hydrogen bond with the phenolate oxygen of PCP in PcpR, while the corresponding residue in TcpR is replaced by the hydrophobic residue L110. In addition, the residue L110 in PcpR, which is located in a hydrophobic core between IBC1 and IBC2, is replaced with the polar residue Q116 in TcpR. Thus it is likely that the orientation of inducer is quite different between these two related transcription factors. Supporting this, PCP does not display any transcriptional effect with TcpR [8], indicating PCP might have affinity only for PcpR and not TcpR. Additional *in vivo* experiments demonstrated PcpR is responsive to several polychlorophenols for activating the expression of the *pcp* genes [4]; however, TcpR is only responsive to 2,4,6-TCP and 2,4,6-tribromophenol, but not other polychlorphenols [8].

A comparison of the PcpR IBC1 residues with those in DntR reveals that S104 of PcpR is replaced with threonine, T104. The hydrogen bond between PcpR-S104 and the phenolate oxygen of PCP is replaced by a water-mediated hydrogen bond between DntR-T104 and salicylate [13]. Other differences in IBC1 between PcpR and DntR include changes of F106I, L110Q, F151L, E170H, and the replacement of V275 in PcpR with I272 in DntR. Close comparison of the residues between PcpR and BenM also illustrates the conservation of hydrophobic character in the binding pockets, while there is little overlap in polar residues.

Another noticeable residue in IBC1 is H206 that is conserved in PcpR, TcpR, and DntR. The imidazole Nε of H206 is hydrogen bonded to the phenolate oxygen of 2,4,6-TCP in PcpR. In contrast, H206 is moved away from the cavity interior in the PCP complex structure, likely due to steric effects from the bulkier PCP inducer. In DntR, the imidazole side chain of H206 forms a hydrogen bond with its inducer molecule, salicylate [13]. In BenM, the corresponding residue is replaced by N202 that is not within contact distance of the inducer molecule.

### 2.5. Substrate Specificity Differences among PcpR, TcpR and Other LTTRs

The PcpR regulation system in *Sphingobium chlorophenolicum* L-1 is activated by several polychlorophenols including PCP, 2,4,5,6-TeCP, 2,4,6-TCP, 2,6-DiCP, and 2,4,6-tribormophenol. On the contrary, the TcpR system in *Cupriavidus necator* JMP134 can be only activated by 2,4,6-TCP and 2,4,6-tribromophenol [8]. The *tcp* genes are likely involved in the degradation of tribromophenol, which is common in the soil and ocean [8,18].

Our ITC data indicated that the IBC1 of PcpR has a higher affinity toward 2,4,6-TCP (*Kd* = 22.8 nM) than PCP (*Kd_1_* = 110 nM). The difference in those *Kd* values resulted from both enthalpic and entropic contributions. The enthalpic contributions of 2,4,6-TCP and PCP binding were −11.9 and −6.9 kcal/mol, respectively, contributing to a large magnitude of the *Kd* difference.

**Figure 7:**
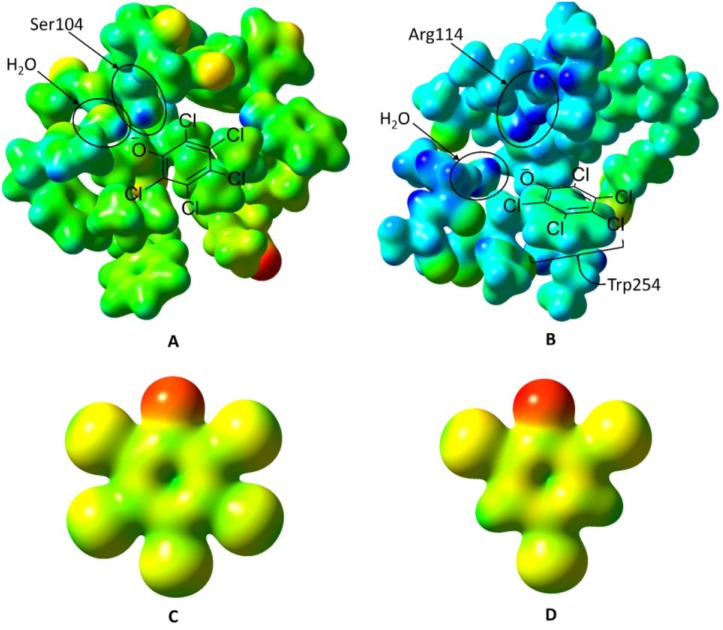
Electrostatic potential surfaces of PcpR’s two ligand-binding sites, pentachlorophenol, and 2,4,6-trichlorophenol. The electrostatic potential for Figures 7A through Figure 7D were all mapped on a scale of −0.250 hartrees (red) to +0.300 hartrees (blue). (**A**) The inducer-binding site 1 (IBC1) of PcpR with His206 in the PCP-bound conformation. Ser104 and the water molecule that donates a hydrogen bond to PCP are indicated. The approximate PCP binding mode is shown as a ChemDraw (Version 14, CambridgeSoft, Cambridge, MA, USA) figure overlaid onto the electrostatic potential surface; (**B**) The inducer-binding site 2 (IBC2) of PcpR in the PCP-bound conformation. Arg114 and the water molecule that donates a hydrogen bond to PCP are indicated. The approximate PCP binding mode is shown as a ChemDraw figure overlaid onto the electrostatic potential surface; (**C**) The electrostatic potential surface of the pentachlorophenolate; and (**D**) The electrostatic potential surface of the 2,4,6-trichlorophenolate anion.

Considering the *pKa* values at 25 °C for 2,4,6-TCP and PCP are 6.1 and 4.5 respectively [23], the hydroxyl group of both molecules at physiological pH should be in a deprotonated state, and ultimately be able to interact with the S104 side chain in IBC1 (Figure 7A). The more hydrophobic nature and larger molecular volume of PCP (Figure 7C) compared to those of 2,4,6-TCP (Figure 7D) could provoke large hydrophobic interaction freeing more water molecules of binding pockets resulting in entropic gain. Neither phenol nor benzoic acid displayed any significant affinity (Figure 5) indicating the contribution of the chlorine groups. In addition, considering the negative entropy in PCP-binding to IBC2 (−11.0 cal·mol^−1^·K^−1^), a substantial change in local conformation should follow upon association, which 2,4,6-TCP cannot invoke due to its relative hydrophobic nature compared to PCP (Figure 7C,D).

The ITC data showed a second binding event for PCP. The second binding site for PCP had a *Kd_2_* = 70 µM, Δ*H*_2_ = −9.0 kcal/mol, and Δ*S*_2_ = −11.0 cal·mol^−1^·K^−1^. This second binding event observed in ITC measurements correlates well with observed PCP at IBC2 in our complex crystal structures. The relatively higher entropic cost of this second binding event is consistent with a substantial rearrangement of the constituent side chains of IBC2.

### 2.6. Oligomerization and Regulation Mechanism of PcpR

Although the asymmetric unit of the PcpR inducer-binding domain was tetrameric, the interactions observed in the inter-dimer interface were relatively weak and the MALS data clearly indicated a dimeric nature (Figure 3). To further analyze the biological relevance of the oligomeric state of PcpR observed in the crystal lattice, the apo-form coordinates were uploaded to the Protein Interfaces, Surfaces, and Assemblies server (PISA) [24]. The results of PISA analysis gave average buried surface area of 1985.3 Å^2^ and Δ*G* of dimer formation to be −10.1 kcal/mol. The complexation significance score (CSS) for this dimer interface was 1. Examination of another interface, could establish a potential tetramer, gave an average buried surface area of 254.1 Å^2^, Δ*G* of interface formation to be −0.25 kcal/mol, and CSS of 0. Overall PISA results suggested the observed dimer to be biologically relevant, but not for tetramer. Thus, the observed tetrameric nature in the asymmetric unit is likely due to crystal packing forces. As indicated in conflicting reports, and various forms of tetramers among inducer-binding domain crystal structures [13], it is highly plausible that inducer-binding domains may not form physiologically relevant tetramers without the presence of the linker helix or DNA-binding domain.

In general, it is believed that specific interactions between LTTR proteins and their corresponding inducer molecules, which is one-to-one in most cases, results in the required conformational changes for oligomerization of the protein [16]. Although two inducer molecules per LTTR subunit has been observed in DntR and BenM, the significance of two-IBC systems in transcriptional regulation is not well understood. Both our crystal structure and ITC data indicated that PcpR-IBC2 showed a weak affinity to PCP, no affinity for 2,4,6-TCP, and a drastically different location compared to that of BenM. 

Considering the close proximity between IBCs, it is plausible to expect a progressive change upon initial high affinity binding to the IBC1 (*Kd* of 110 nM) and followed by the low affinity binding to IBC2 (*Kd* of 70 µM), and thus a full range of conformational change could occur only when both sites are occupied. Supporting this speculation, the complete conformational change of BenM occurs only in the event of both IBC1 and IBC2 being occupied [14]. In the case of *Acinetobacter baylyi*, its operon is activated upon binding of either benzoate or *cis*–*cis* muconate onto BenM and its full transcriptional activation requires the presence of both inducer molecules [13,17]. It has also been reported that the fully closed conformation of DntR from *Burkholderia cepacia* R34 can only be established when both IBC1 and IBC2 are occupied [13]. However considering the two facts that 2,4,6-TCP can occupy only IBC1 of PcpR, but both PCP and 2,4,6-TCP are the effective translational regulator for PcpR, the significance of IBC2 and progressive induction in PcpR is doubtful.

Despite the close proximity of the PCP-binding sites in PcpR, there were no significant conformational changes of the backbone, or the dimer orientations upon comparing the apo-form to either the PCP or 2,4,6-TCP complex structures. Comparison of PcpR with BenM and DntR [13] indicates that both our apo-form and inducer bound structures of PcpR are in the closed conformation, which could be due to a crystal packing forces. However, the structure of PcpR in solution could adopt an open conformation and inducer-binding triggers a transition to a closed form and this dynamic motion of the inducer-binding domain between an open and closed conformation could be communicated to the DNA binding domain via the linker helix as suggested in previous models to effect a transcriptional response [12,13].

## 3. Experimental Section

### 3.1. Chemicals

Chemicals were obtained from Sigma Aldrich (St. Louis, MO, USA) or Fisher Scientific (Waltham, MA, USA). Crystallization screens were obtained from Hampton Research (Aliso Viejo, CA, USA).

### 3.2. Cloning, Expression and Purification

The *pcpR* gene that covers a substrate-binding domain (residues from 85 to the *C*-terminus of PcpR) was cloned into the pET30-LIC vector, and expressed as a *C*-terminal HIS6X fusion protein. Two hundred milliliters of luria broth (LB) supplemented with 30 μg/mL kanamycin was inoculated with a freezer stock of pET30a PcpR in BL21(DE3) cells and incubated overnight at 37 °C with constant shaking at 250 rpm. The 200 mL culture was then used to inoculate 4.5 L of LB medium. Protein expression was induced by addition of isopropyl β-d-thiogalactopyranoside (IPTG) to 0.5 mM final concentration at mid-log phase (*A*_600_ = ~0.6). Following induction, the cells were further incubated for 12 h at 20 °C with constant shaking at 200 rpm. The cells were then harvested by centrifugation (3000× *g*), after which the pellet was frozen to promote cell lysis. The pellet was thawed at room temperature and suspended in a minimal volume of lysis buffer (50 mM Tris pH 8.0, 300 mM NaCl, 20 mM imidazole and 5% *v*/*v* glycerol). The cell suspension was sonicated (450 Sonifier^®^, Branson Ultrasonics, Danbury, CT, USA), and the resulting lysate cleared by centrifugation (20,000 × *g* for 20 min).

Lysate was applied to a nickel-nitrilotriacetate column and washed with several column volumes of lysis buffer. Elution buffer consisted of lysis buffer supplemented with 250 mM imidazole. Eluted fractions containing PcpR were combined, concentrated, and buffer-exchanged into 20 mM Tris (pH 8.5) by ultrafiltration in an Amicon 8050 cell (Millipore, Billerica, MA, USA) with a 5-kDa cutoff polyethersulfone membrane (Millipore), loaded onto a Mono Q™ GL10/100 anion-exchange column (GE Healthcare, Piscataway, NJ, USA), and eluted at 200 mM NaCl with a linear NaCl gradient of 0 to 2 M NaCl using a preparative HPLC (Akta Explorer, GE Healthcare). Fractions containing PcpR were pooled, concentrated, and exchanged into 20 mM Tris (pH 8.5) with 5% *v*/*v* glycerol. Final homogeneity of the purified proteins was estimated over 99%. Purity was monitored for all protein preparations by SDS-PAGE and protein concentrations were determined by Bradford assay using BSA as a standard.

### 3.3. Protein Crystallization and Structure Determination

Crystals of PcpR were grown using the hanging drop vapor diffusion method. Purified PcpR (10 mg/mL) in 20 mM Tris pH 8.5 with 5% *v*/*v* glycerol was mixed with an equal volume of reservoir solution and equilibrated against the same solution at 4 °C. The reservoir solution for PcpR apo-form crystals was 0.2 M sodium tartrate dibasic dihydrate with 20% *w*/*v* polyethylene glycol 3350. Crystals of PcpR typically appeared within 10–15 days. PcpR:PCP complex crystals were obtained by first growing apo-form crystals in a different condition (0.1 M sodium malonate pH 5.0 with 12% *w*/*v* polyethylene glycol 3350). These crystals were subsequently soaked with PCP and incubated for 30 min.

Co-crystals of PcpR complexed with 2,4,6-TCP were grown using the hanging drop vapor diffusion method just as with the apo-crystals of PcpR except the purified PcpR was first buffer exchanged to 20 mM Tris, 100 mM NaCl pH 7.5 with 5% *v*/*v* glycerol. The reservoir condition used for crystallization was 0.2 M sodium tartrate dibasic dihydrate, 20% *w*/*v* PEG 4,000. The hanging drop was set with 2 µL of PcpR (8.4 mg/mL), 2 µL reservoir solution, and 2 µL of either 2,4,6-TCP (1.5 mM) or PCP (1.5 mM). Both PcpR-2,4,6-TCP and PcpR-PCP co-crystals grew in 2 to 3 days.

All crystals were cryoprotected during looping using reservoir solution supplemented with of 15% *v*/*v* glycerol and subsequently flash frozen in liquid nitrogen. All diffraction data were collected at the Lawrence Berkeley National Laboratory Advanced Light Source (ALS, beam line 8.2.1, Berkeley, CA, USA) and processed with the HKL2000 package [25]. The statistics for the diffraction data are listed in Table 1. Initial phasing of apo-form PcpR diffraction data was conducted by molecular replacement with the PDB coordinates, 2Y7W, using PHENIX phaser [26]. Iterative model building and refinement took place using the programs COOT [27] and PHENIX [26]. The PcpR apo-form model was used for initial phasing of the complex data sets described above.

### 3.4. Isothermal Titration Calorimetry (ITC)

Isothermal titration calorimetric reactions were carried out on a MicroCal iTC200 instrument (GE Healthcare). The PcpR inducer domain was prepared for ITC by buffer exchange into 20 mM Tris, 100 mM NaCl, pH 7.5 at 4 °C. PcpR protein in the calorimetric reaction cell was diluted to 50 µM with a 20 mM Tris, 100mM NaCl, 1% DMSO, pH 7.5. All titrations were performed at 25 °C with a stirring speed of 750 rpm with an initial injection of 0.8 µL and 25 subsequent injections of 1.5 µL. Ligands were diluted into the buffer used for protein buffer exchange and injected into the MicroCal iTC200 sample cell containing protein solution, and the heats of binding were recorded. Ligands were also titrated against buffer to account for the heats of dilution. Ligand concentrations were adjusted to obtain significant heats of binding, and the time intervals between injections were also adjusted to ensure proper baseline equilibration. Origin 7 Microcal Data Analysis software analysis package (GE Healthcare) was used for ITC curve fitting. A two set of sites model was employed. Curve fitting equations can be found in the Microcal ITC200 User Manual appendix [28]. 

### 3.5. Multi-Angle Light Scattering (MALS)

The weight-average molecular mass of PcpR was measured by combined size exclusion chromatography and multi-angle laser light scattering as described previously [29]. Briefly, 200 μg of PcpR was loaded onto a BioSep-SEC-S 2000 column (Phenomenex, Torrance, CA, USA) and eluted isocratically with a flow rate of 0.5 mL·min^−1^. The eluate was passed through a tandem UV detector (Gilson, Middleton, WI, USA), Optilab digital signal processing interferometric refractometer (Wyatt Technology, Santa Barbara, CA, USA), and a Dawn EOS laser light scattering detector (Wyatt Technology). Scattering data was analyzed using the Zimm fitting method with the Astra software package (Wyatt Technology).

### 3.6. Electrostatic Potential Surface Calculation of Ligands and Binding Pocket

Pentachlorophenolate anion and 2,4,6-trichlorophenolate anion were optimized in Gaussian 09 [30] at the B3LYP level of theory using augmented triple-zeta correlation-consistent basis sets (aug-cc-pVTZ) [31]. Total electron density and electrostatic potential grids at 12 points per bohr were generated from the optimized molecular self-consistent field densities using the Gaussian 09 cubegen utility and mapped in GaussView 3.09 [32] as the electrostatic potential on the electron density at 0.020 electrons/bohr^3^.

The electrostatic potential surface of the IBC1 and IBC2 were generated by performing a quadratically-convergent single-point calculation at the B3LYP/cc-pVDZ level of theory (aug-cc-pVDZ for N and O) on select residues taken from the PcpR:PCP complex structure. Idealized hydrogen atoms were added to the structure using the Phenix ReadySet utility [26]. Self-consistent field total electron density and electrostatic potentials were generated at 12 and 6 points per bohr, respectively, and plotted using the same method as described for PCP and 2,4,6-TCP. All electrostatic potentials were plotted on a range of −0.250 hartrees (red) to +0.300 hartrees (blue).

## 4. Conclusions

The work presented here illustrates the PcpR transcriptional control system that regulates the expression of the dechlorination enzymes responsible for PCP degradation. PcpR has two inducer binding cavities (IBCs), both of which contain the inducer molecule, PCP; however, the affinity of IBC1, is much higher than that of IBC2. Multiple binding sites have been noticed across the LysR transcription family, however, the placement of the second binding site is quite varied and its significance in transcriptional control is not well understood. Considering the fact that another effective inducer, 2,4,6-TCP, has no affinity to the IBC2, binding to IBC1 is likely sufficient to turn on the transcription of participating genes. When PcpR is compared with TcpR, most of the residues constituting the inducer-binding cavities are different, suggesting the two regulator proteins should have evolved a different inducer-binding mode and specificity. This finding is in agreement that PcpR uses several polychlorophenols, including PCP, 2,4,6-trichlorophenol and 2,4,6-tribromoophenol, as inducers; whereas TcpR is only responsive to 2,4,6-trichlorophenol and 2,4,6-tribromoophenol.
